# Possible Involvement of Cone Opsins in Distinct Photoresponses of Intrinsically Photosensitive Dermal Chromatophores in Tilapia *Oreochromis niloticus*

**DOI:** 10.1371/journal.pone.0070342

**Published:** 2013-08-05

**Authors:** Shyh-Chi Chen, R. Meldrum Robertson, Craig W. Hawryshyn

**Affiliations:** 1 Department of Biology, Queen’s University, Kingston, Ontario, Canada; 2 Centre for Neuroscience Studies, Queen’s University, Kingston, Ontario, Canada; University of Oldenburg, Germany

## Abstract

Dermal specialized pigment cells (chromatophores) are thought to be one type of extraretinal photoreceptors responsible for a wide variety of sensory tasks, including adjusting body coloration. Unlike the well-studied image-forming function in retinal photoreceptors, direct evidence characterizing the mechanism of chromatophore photoresponses is less understood, particularly at the molecular and cellular levels. In the present study, cone opsin expression was detected in tilapia caudal fin where photosensitive chromatophores exist. Single-cell RT-PCR revealed co-existence of different cone opsins within melanophores and erythrophores. By stimulating cells with six wavelengths ranging from 380 to 580 nm, we found melanophores and erythrophores showed distinct photoresponses. After exposed to light, regardless of wavelength presentation, melanophores dispersed and maintained cell shape in an expansion stage by shuttling pigment granules. Conversely, erythrophores aggregated or dispersed pigment granules when exposed to short- or middle/long-wavelength light, respectively. These results suggest that diverse molecular mechanisms and light-detecting strategies may be employed by different types of tilapia chromatophores, which are instrumental in pigment pattern formation.

## Introduction

The ability to detect and respond to the visual environment plays a critical role in the survival of animals. Changing the body color pattern is a strategy frequently used by lower vertebrates and invertebrates, allowing them to respond to biotic and abiotic stimuli. These color-changing mechanisms greatly impact physiological and behavioural aspects of animals, including UV protection [1], [2], thermoregulation [3], [4], concealed communication [5], [6], camouflage [7], [8], mate choice [9], [10], aggressive signaling [11], [12], and social status display [13], [14]. Chromatophores, dermal specialized pigment cells, are thought to be the primary agents that shape body patterns in many animals. Chromatophores are typically categorized into six classes based on their internal structure and pigment colors: melanophores, erythrophores, xanthophores, cyanophores, leucophores, and iridophores [15]–[17]. They are capable of responding morphologically (in number or size) or physiologically (through translocation of inner pigment granules, i.e. aggregation or dispersion) in order to execute long-term or immediate color pattern changes, respectively [18], [19]. Integumentary color change is achieved in three ways: 1) through pigment granule motility, 2) through reflective plates within chromatophores, and 3) through coordination of different chromatophore classes and/or other tissues [20], [21]. The translocation of pigment granules within chromatophores was shown to be mediated by the action of microtubules and microfilaments, as well as various molecular motors [22], [23]. In teleosts, this translocation is controlled by the sympathetic nervous system [24], [25] and regulated by hormones such as prolactin, catecholamines, melatonin, noradrenaline (NA), melanin-concentrating hormone (MCH), and alpha-melanocyte-stimulating hormone (α-MSH) [9], [14], [26]–[30]. For example, erythrophores in Nile tilapia (*Oreochromis niloticus*) and swordtail (*Xiphophorus helleri*), pigment aggregation can be triggered by MCH, while dispersion can be mediated by MSH [28], [29]. Pharmacological evidence suggests that the increase or decrease in intracellular cAMP level regulated by G_s_ or G_i_ protein plays an important role in signal transduction pathways of hormone-mediated pigment granule movements in chromatophores [29], [31].

Recently, studies have shown that light can also trigger the color-changing process, and that these photoresponses depend on the intensity and wavelength of the light stimulus [32]–[35]. Chromatophore spectral sensitivity has been measured from several teleosts. For example, the most effective wavelength to induce melanophore dispersion of *Oryzias latipes* and *Zacco temmincki* are near 415 and 525 nm, respectively [36], [37]. These results show that maximum chromatophore photosensitivity varies across species. Moreover, the light-induced translocation of intracellular pigment granules (i.e. retrograde vs. anterograde) is not identical in the same chromatophore class. For instance, xanthophores under illumination disperse in *Trematomus bernacchii*, but aggregate in *Oryzias latipes* [38], [39].

Opsins have been suggested to be involved in chromatophore photoresponses, as putative opsins have been detected in skin tissues 32,33,40. However, this hypothesis relies mainly on molecular evidence, e.g. opsin gene expression in integumentary tissues [32], [33]. Moreover, chromatophore photoresponses were proposed to be generated through a pathway other than the G_t_ protein-activated cGMP signaling pathway in retinal photoreceptors. Thus, chromatophore dispersion and aggregation were suggested to occur through the G_s_ or G_i_ protein-activated cAMP signal transduction pathway, similar to that found in hormonal regulation of chromatophores [31], [32], [41]. Nevertheless, the relationship between visual pigments and chromatophore photoresponses remains unclear.

An opsin protein in combination with a chromophore forms a visual pigment. Different classes of opsins vary in amino acid sequences leading to the change of spectral absorption properties of visual pigments. In vertebrates, five classes of retinal opsin genes have been identified: one rod opsin (*RH1*) and four classes of cone opsins including short wavelength sensitive 1 (*SWS1*), short wavelength sensitive 2 (*SWS2*), rod opsin-like (*RH2*), and long wavelength sensitive (*LWS*) opsins [42]. Due to gene duplication, Nile tilapia (*Oreochromis niloticus*) possesses 7 cone opsin genes with distinct peak absorbances (λ_max_): *SWS1* (360 nm), *SWS2b* (423 nm), *SWS2a* (456 nm), *RH2b* (472 nm), *RH2aβ* (518 nm), *RH2aα* (528 nm), and *LWS* (561 nm) [43]. In the present study, we investigated the relationship between cone opsin expression and tilapia chromatophore photoresponses. Using RT-PCR, cone opsin expression was detected in tilapia caudal fin and co-expression of different opsin classes was found in individual melanophores and erythrophores at the single-cell level. We also showed these two major types of pigmented chromatophores (melanophores and erythrophores) in tilapia caudal fin demonstrated distinct photoresponses to incident light stimuli. Under illuminations, melanophores extended their processes and maintained dispersions by shuttling pigment granules (melanosomes). On the other hand, erythrophores aggregated and dispersed pigment granules (erythrosomes) at different spectral range. We suggest that this co-expression of different opsin classes is correlated to the dynamic photoresponses of these intrinsically photosensitive dermal chromatophores (ipDCs) in Nile tilapia. These findings are significant to our understanding of light-driven mechanisms within specific chromatophore classes, and advance our knowledge of how intrinsic dermal photosensitivity contributes to organismal survival and interaction.

## Materials and Methods

### Ethics Statement

The protocol used for the experimental fish was reviewed and approved by the Queen’s University Animal Care Committee (Protocol NO: Hawryshyn-2010-004-R3-A1) and all procedures complied with the Canadian Council for Animal Care regulations.

### Animal

**Figure 1:**
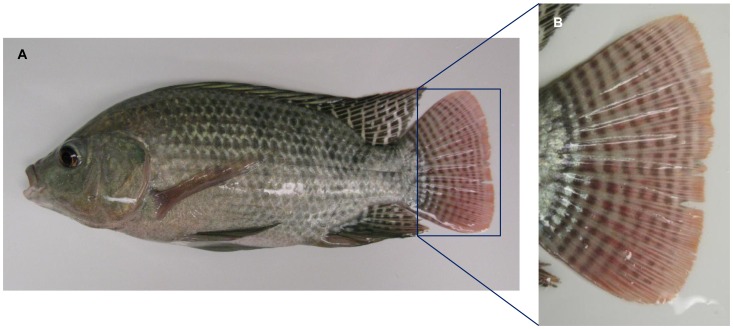
An adult male Nile tilapia *Oreochromis niloticus* (A) and a close-up of the caudal fin (B) showing pigmentation traits.

**Figure 2:**
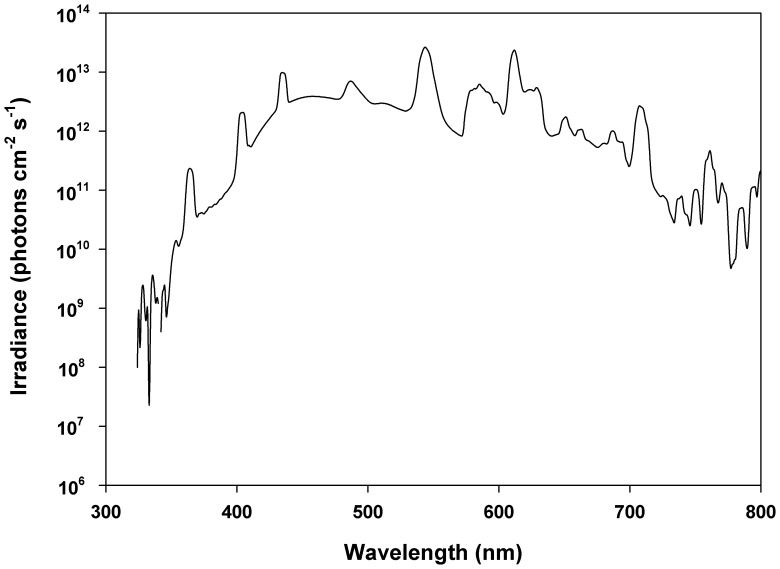
Spectral irradiance in fish culture facility.

Adult male Nile tilapia *Oreochromis niloticus* (Figure 1A; 46.7±7.1 g body mass, 14.8±0.6 cm standard length) were obtained from a local fish farm, Northern American Tilapia Inc. (Lindsay, Ontario, Canada). Fish were kept at a water temperature of 25°C under a 12 h:12 h L:D light cycle and the lighting condition was provided by full spectrum fluorescent lamps (see Figure 2; Full Spectrum Solutions, Inc., Jackson, MI, USA). Fish were anaesthetized by immersion in MS-222 (Syndel Laboratories Ltd., Qualicum Beach, BC, Canada) and sacrificed by cervical transection. Because circadian changes of opsin expression and pigmentary patterns have been reported in fish [44], [45], dissections were always carried out from 11∶00 am to 13∶00 pm. All procedures complied with the Canadian Council for Animal Care regulations and the Queen’s University Animal Care Committee.

### Preparation of Split-fin Tissues and Isolation of Single Chromatophore

Chromatophores were isolated by methods described previously [46]–[48]. Briefly, the integumentary tissues were excised, rinsed with 70% ethanol as well as Ca^2+^, Mg^2+^-free, Dulbecco’s phosphate-buffered saline (CMF-PBS: NaCl 136.9 mM, KCl 2.7 mM, Na_2_HPO_4_ 8.1 mM, KH_2_PO_4_ 1.5 mM; pH 7.2), and cut into pieces about 5-mm^2^ in CMF-PBS. To remove the epidermis, tissues were incubated in EDTA-bicarbonate solution (pH 7.4) and stirred for 20 min. Following incubation with vigorous shaking in 0.25% collagenase type II (Sigma-Aldrich, St. Louis, MO, USA) for 30 min, chromatophores were isolated from split-fin tissues. The dissociated cells were filtered through 140-µm Nylon membrane filter (Millipore, Billerica, MA, USA) and suspended in CMF-PBS. Specific classes of chromatophores were identified and selected under a dissecting microscope (Nikon Instruments Inc., Melville, NY, USA; SMZ1500) equipped with an epi-illumination system (Dolan-Jenner Industries, Boxborough, MA, USA) and polarizer (Nikon Instruments Inc., Melville, NY, USA; MNN40920).

### RNA Extraction, and First Strand cDNA Synthesis

Total RNAs were extracted from caudal fins and single chromatophores (isolated from caudal fins) using Absolutely RNA Miniprep and Nanoprep Kits (Stratagene, La Jolla, CA, USA), respectively. Reverse transcriptions were performed by SuperScript III First-Strand Synthesis SuperMix for qRT-PCR (Invitrogen Canada, Burlington, ON, Canada), following the manufacturer’s manual. To determine if genomic DNA contamination existed in the sample, reverse transcription for non-RT control was conducted with all reagents, except the reverse transcriptase.

### RT-PCR Analysis

Primers for tilapia cone opsins were designed using Primer 3 software based on the sequences published on GenBank (refer to Table 1) and used in RT-PCR, and single-cell RT-PCR analysis. All of the primers were tested in PCR using GoTaq Flexi DNA Polymerase (Promega, WI, USA) in Eppendorf Mastercycler gradient (Eppendorf Canada, Mississauga, ON, Canada) under the same conditions [92°C, 2 min; 92°C, 30 s, 60°C, 30 s, 72°C, 30 s (40 cycles)] prior to being used in subsequent experiments. PCR products were sequenced (McGill University and Genome Quebec Innovation Centre, Montreal, Quebec, Canada) and verified by comparison with corresponding sequences on GenBank. Except cone opsins, we included *rhodopsin* (*RH1*), *melanopsin* (homolog of zebrafish *OPN4a*), and *teleost multiple tissue* (homolog of pufferfish *TMT*) *opsin* in single-cell RT-PCR analysis and no expression was detected in individual chromatophores examined (data not shown; n = 7 for melanophores, and n = 12 for erythrophores).

**Table 1:** Primers used in RT-PCR assays.

Gene	Accession no. (GenBank)	Forward Primer (5′→3′) Reverse Primer (5′→3′)	Amplicon size (bp)
*SWS1*	AF191221	TCCACCTGTACGAGAACATCTCCAA GGTGTGCCAGCAAACAGGACAA	124
*SWS2b*	AF247120	CAAGAAGCTCCGGTCTCATC ATGCAGTTGGACCAAGGAAC	134
*SWS2a*	AF247116	CGCTCGGTAACTTTGCTTTC AGCACTGTAGGCCTTCTGGA	132
*RH2b*	DQ235681	CTGGTCACCGCTCAAAACAA TCAAAGGACCCAAGGAGAAATAG	148
*RH2aβ*	DQ235682	CACCATCACAATCACGTCTGCTAT CCAGGACAACAAGTGACCAGAG	122
*RH2aα*	DQ235683	CCATCACCATCACATCAGCTG CCAGGACAACAAGTGACCAGAG	120
*LWS*	AF247128	TCATCTCCTGGGAAAGATGG TCCAAATATGGGAGGAGCAC	134
*RH1*	AY775108	ATATGTTGGCTGCCCTATGC TGCTCCCTCCTCTTCTTCAA	216
*OPN4*	GR605566	ACTGCACTGAGCACCATCAC TAGATGACCGGAGCATTTCC	196
*TMT*	AF402774	CCGTCCAACTACTGCAAGGT CACGATCAGGCAGAAGACAAA	198
*β-actin* a	EF206796	TGCGTGACATCAAGGAGAAG CTCTCGTTCCCAATGGTGAT	136

a *β-actin* was used as a positive control in RT-PCR assays.

To investigate cone opsin expression profiles in different types of chromatophores, single-cell RT-PCR was conducted under the aforementioned condition and a power analysis (statistical power = 0.9, α = 0.05, two-tailed test) was administrated to determine the sample size of cells (n = 22 for melanophores, n = 28 for erythrophores).

### Measurements of Chromatophore Photoresponses

Split-fin tissues containing chromatophores were incubated in the culture medium (mixture of Leibovits L15 medium, fetal calf serum, and water in a ratio 80∶15∶5, penicillin-G 100 U/ml, kanamycin 100 µg/ml) at 25 °C in a water-jacked CO_2_ incubator in the dark for 2 days. After 2 days of culture, tissues were immersed in PBS for 15 min before experiments. Light stimuli were generated by a 150 W xenon lamp system and a monochromator (Photon Technology International, London, ON, Canada). To measure photoresponses of melanophores and erythrophores, individual chromatophores were challenged with equal-quanta spectral irradiance (13.9 log photons cm^−2^ s^−1^) and 10-nm FWHM values (Full Width at Half Maximum) at one of the stimulating wavelengths (380, 420, 460, 500, 540, and 580 nm) for 3 minutes. Images of chromatophore photoresponses were taken using a Qimaging Microimager II CCD camera and QCapture Suite V2.46 software (Qimaging, Burnaby, BC, Canada). All the experiments were conducted at 25°C in darkness and tissues were continuously perfused with PBS.

## Results

### Cone Opsin Gene Expression in Tilapia Caudal Fin

**Figure 3:**
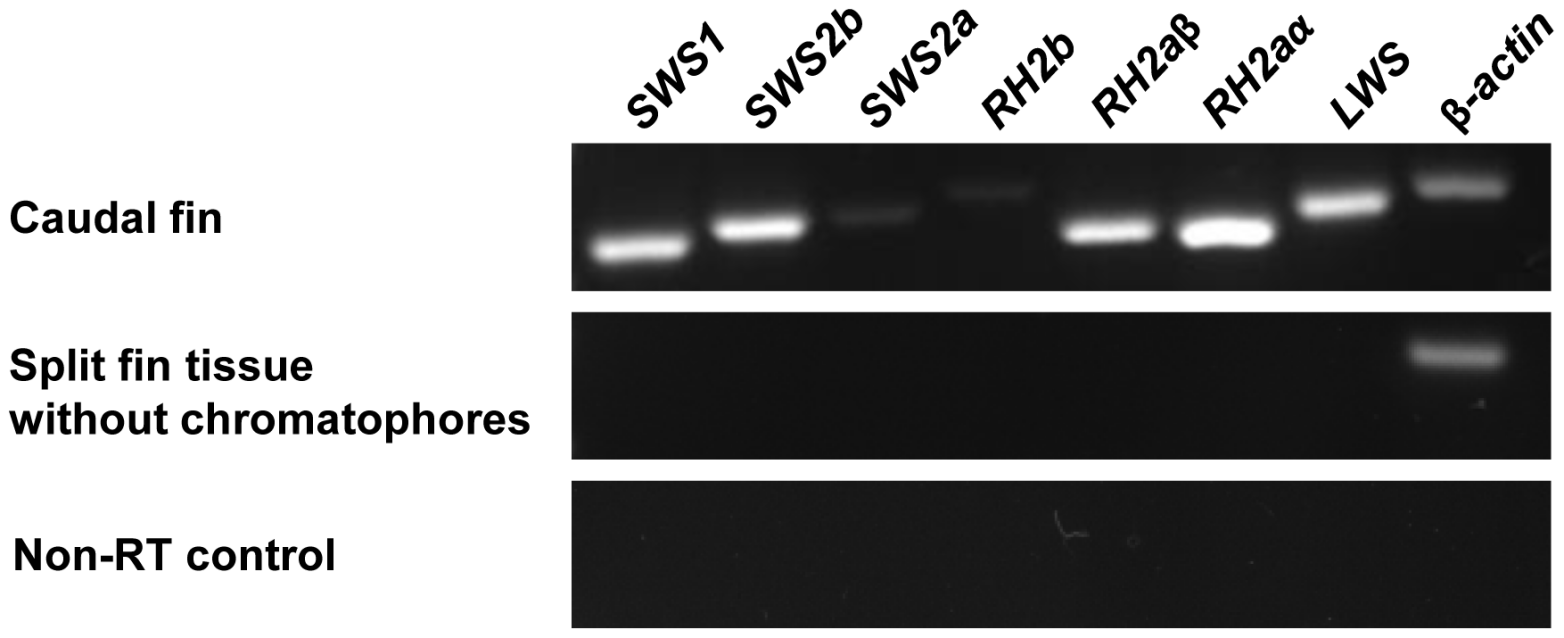
Cone opsin expression in tilapia caudal fin. Using RT-PCR, 7 tilapia cone opsins were detected in tilapia caudal fin but not in the split fin tissue without chromatophores. No detectable signal was found in the non-RT control sample. *β-actin* was used as a positive control in RT-PCR analysis.

In sexually mature male tilapia, caudal fins typically bear pigmentation traits such as vertical bars (Figure 1B); these bars contain a high density of chromatophores. Cone opsin expression in tilapia caudal fin was investigated using RT-PCR with primer sets amplifying the target tilapia cone opsin genes (see Table 1). All cone opsin genes were detected in caudal fin but not in the split fin tissue without chromatophores (Figure 3). Therefore, we suggest cone opsin expression in tilapia caudal fin could be associated with the presence of chromatophores.

### Cone Opsin Expression of Individual Melanophores and Erythrophores

To confirm cone opsin expression in individual chromatophore, we performed single-cell RT-PCR on melanophores and erythrophores. Opsin gene classes expressed in individual melanophores and erythrophores varied in number (Table 2 and 3). The results showed that co-expression of different classes of cone opsin genes was frequently found in chromatophores we examined (73% in melanophores and 64% in erythrophores). Among cells expressing only one opsin, melanophores expressed either *SWS1* or *RH2b* (Table 2); erythrophores expressed *SWS1* or one of the *RH2* group genes (*RH2b*/*RH2aβ*/*RH2aα*; Table 3). When expressing more than one class of opsins, most melanophores expressed *SWS1* with other class of opsin genes (14 out of 16) and erythrophores tended to co-express *SWS1* with at least one of the *RH2* group genes (*RH2b*/*RH2aβ*/*RH2aα*) (17 out of 18). The opsin expression profiles from melanophores and erythrophores suggested that *SWS1* and *RH2* genes are primarily expressed genes and may play important roles in chromatophore photosensitive functions.

**Table 2:** Cone opsin expression profile of tilapia melanophores.

Cell No	Cone opsin							Number of opsin classes detected per cell
	*SWS1*	*SWS2b*	*SWS2a*	*RH2b*	*RH2aβ*	*RH2aα*	*LWS*	
**1**	+	+						2
**2**	+							1
**3**	+			+				2
**4**	+						+	2
**5**				+		+	+	3
**6**				+				1
**7**	+				+		+	3
**8**				+			+	2
**9**	+			+				2
**10**	+			+				2
**11**	+						+	2
**12**				+				1
**13**	+							1
**14**	+	+		+				3
**15**	+					+		2
**16**	+		+					2
**17**	+							1
**18**	+		+					2
**19**	+							1
**20**			+		+		+	3
**21**	+			+	+			3
**22**	+			+	+			3
**Total**	17	2	3	10	4	2	6	

**Table 3:** Cone opsin expression profile of tilapia erythrophores.

Cell No	Cone opsin							Number of opsin classes detected per cell
	*SWS1*	*SWS2b*	*SWS2a*	*RH2b*	*RH2aβ*	*RH2aα*	*LWS*	
**1**	+	+	+	+	+			5
**2**	+							1
**3**	+							1
**4**				+				1
**5**	+			+	+			3
**6**	+				+			2
**7**	+			+	+			3
**8**					+			1
**9**	+			+	+			3
**10**	+							1
**11**	+			+	+			3
**12**						+		1
**13**	+			+				2
**14**	+							1
**15**	+			+				2
**16**	+	+	+	+		+		5
**17**	+		+	+		+	+	5
**18**	+			+		+		3
**19**	+		+	+		+		4
**20**	+	+	+	+	+	+		6
**21**	+	+	+	+	+	+		6
**22**	+	+	+		+	+		5
**23**	+				+		+	3
**24**	+							1
**25**			+		+			2
**26**				+				1
**27**				+				1
**28**	+			+			+	3
**Total**	22	5	8	16	12	8	3	

### Photoresponses of Melanophores and Erythrophores

**Figure 4:**
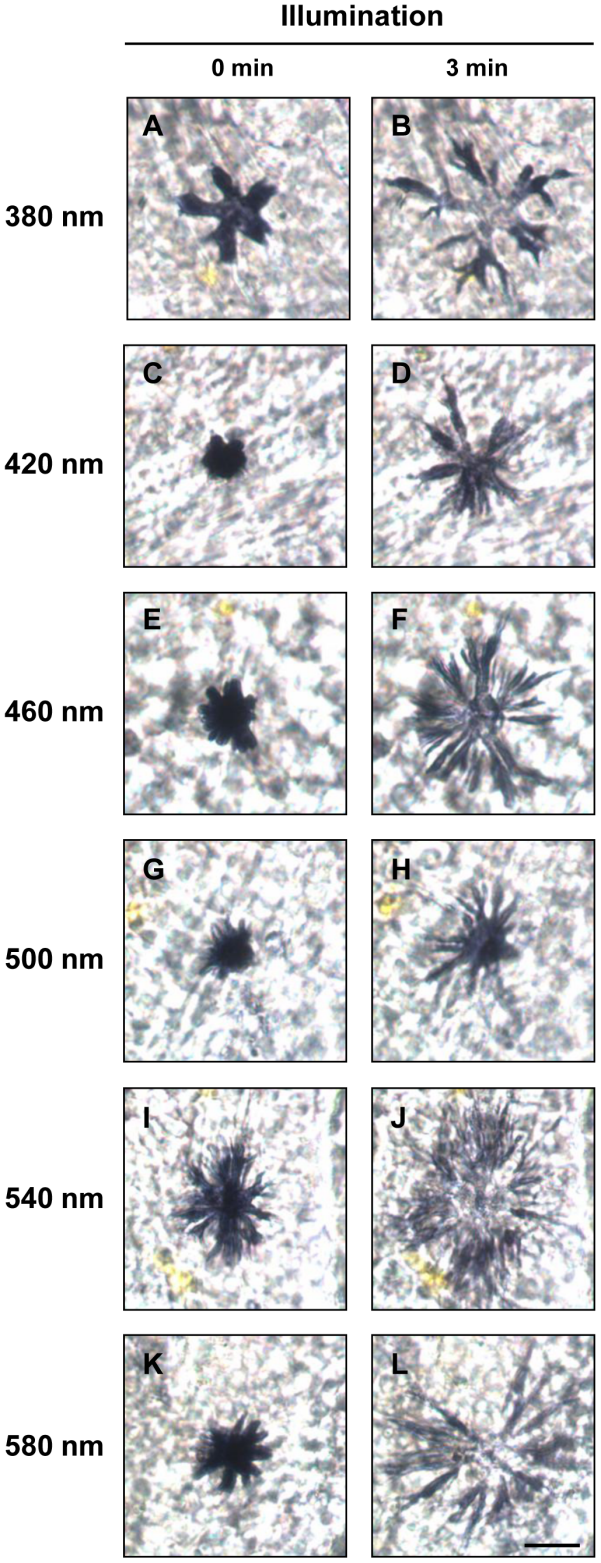
Photoresponses of tilapia melanophores. Individual melanophores were challenged with equal-quanta spectral irradiance (13.9 log photons cm^−2^ s^−1^) at one of the stimulating wavelengths (A,B: 380 nm; C,D: 420 nm; E,F: 460 nm; G,H: 500 nm; I,J: 540 nm; K,L: 580 nm; n = 4 at each test wavelength) for 3 minutes. A,C,E,G,I,K: before illumination; B,D,F,H,J,L: after illumination. Scale bar: 40 µm.

**Figure 5:**
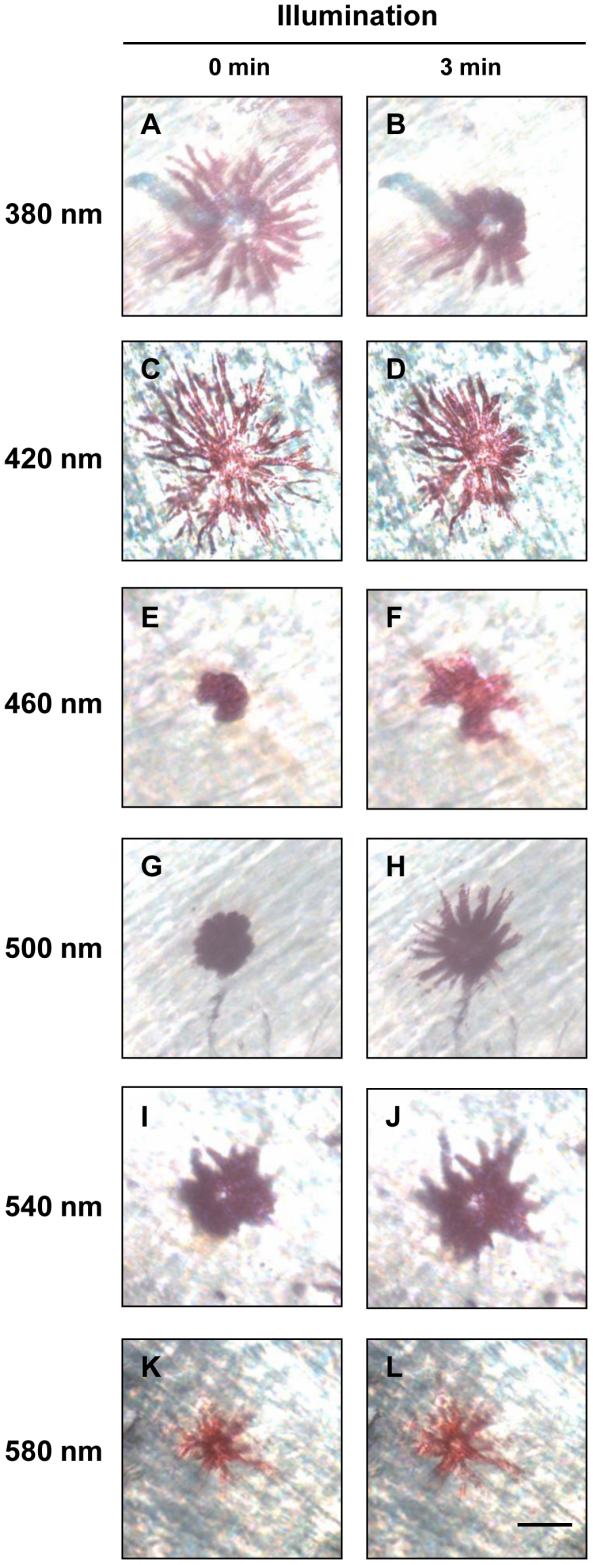
Photoresponses of tilapia erythrophores. Individual erythrophores were challenged with equal-quanta spectral irradiance (13.9 log photons cm^−2^ s^−1^) at one of the stimulating wavelengths (A,B: 380 nm; C,D: 420 nm; E,F: 460 nm; G,H: 500 nm; I,J: 540 nm; K,L: 580 nm; n = 5 at each test wavelength) for 3 minutes. A,C,E,G,I,K: before illumination; B,D,F,H,J,L: after illumination. Scale bar: 20 µm.

In order to understand the relationship between opsin expression and the dynamic chromatophore photoresponse, we measured chromatophore responses at six wavelengths ranging from 380 to 580 nm. Both of melanophores and erythrophores were light-sensitive, but showed their photoresponses in different manners. Regardless of wavelengths, melanophores dispersed and tended to maintain their shape in the dispersion stage by shuttling pigment granules when receiving light stimulus (Figure 4; also see Movie S1). On the other hand, erythrophores exhibited wavelength-dependent photoresponses: aggregations were induced when short-wavelength light was applied (Figure 5A–D), while dispersions occurred when cells were presented with middle- and long-wavelength light (Figure 5E–L).

## Discussion

In previous studies, opsins detected in integumentary tissues are suggested to endow chromatophores with the ability to respond to light. However, the lack of direct evidence showing opsin expression at the single-cell level or the link of opsins and chromatophore photoresponses has hindered our understanding of the photosensitive mechanism within chromatophores. In the present study, cone opsin expression was detected in tilapia caudal fins where chromatophores are present. We further employed single-cell RT-PCR to demonstrate the specific opsin expression profiles in two chromatophore populations. We found *SWS1* is the prominent opsin expressed in both melanophores and erythrophores. In addition, the expression profile reveals that in most erythrophores, *SWS1* is co-expressed with at least one of the *RH2* group genes. When presented with light stimuli at different wavelengths, melanophores and erythrophores showed distinct responses. Under illuminations, melanophores dispersed and shuttled melanosomes to maintain cell shape in the dispersion stage while erythrophores acted in a wavelength-dependent manner. Although a variety of opsin repertoires are present in melanophores and erythrophores, some particular classes of opsins detected at higher frequencies may be responsible for their distinct photoresponses. More functional characterizations of ipDC photoresponses are required to clarify the involvement of opsins in this extraretinal photosensitive system.

In addition to inter- and intraspecific communication, there is also evidence that color changes are closely associated with habitat or background colors [18], [49]. For example, when medaka *Oryzias latipes* are adapted to white background, their body colors become paler due to the aggregation, reduced cell size, or even melanophore apoptosis [19], [50]. These results highlight the importance of environmental photic factors on body color patterns; however, how animals coordinate visual cues with their body color performances remains unclear. Previous work has suggested that chromatophores are able to directly respond to incident light, but this work lacked molecular evidence at the single-cell level to demonstrate that visual pigments are expressed in chromatophores. Studies using spectral analyses show that chromatophores in some vertebrates possibly express only one opsin, which regulates one corresponding photoresponse [36], [37], [51], [52]. More recently, PCR data from tilapia skin tissue but not from single chromatophore, suggested tilapia erythrophores express two putative opsins, *RH2* and *LWS* [32]. In the present study, however, we detected *SWS1* and *RH2* group genes at higher frequency and *LWS* at lower frequency in opsin expression profile of erythrophores (see below and results of single-cell RT-PCR as well). Our results were obtained at the single cell level, which eliminated the possibility of misrepresented expression pattern from mixed cells, or contamination. Moreover, the previous study used primers sets restricted to two opsins, *RH2* and *LWS*. These explain the discrepancy between our work and previous results. Therefore, we suggest molecular work should be conducted at single-cell level to truly reflect the expression file of a specific chromatophore population.

Distinct photoresponse of tilapia melanophores and erythrophores suggest they may have different importance in physiological functions. Melanophores were suggested to prevent incident light from penetrating body surface and harming internal organs since melanin has been thought to provide effective photoprotection via absorption of UVA and UVB [53], [54]. Nevertheless, based on the photoresponses measured herein, melanophores more likely serve as a broad-spectrum light filter. Instead, dynamic photoresponses of erythrophores can generate specific photic effects with other classes of chromatophores in a dermal chromatophore unit when confronting various light stimuli.

Recently, increasing evidence for extraretinal photoreceptors in varied locations across the body has been reported, and their visual pigments are suggested to aid in physiological regulations such as circadian rhythms, pupil size, and body coloration tuning [55]. Indeed, increasing evidence shows that some animals possess light-mediated behaviours due to their dermal sensitivity [56]–[58]. Moreover, evidence shows that *SWS1* opsin expression in pineal organ is detected during different life stages in a wide rage of teleosts [59]. Since high frequency of *SWS1* expression was found in cone opsin expression profiles of tilapia melanophores and erythrophores, ultraviolet photosensitivity may play important roles in some extraretinal photoreception. Although no expression of *RH1*, *OPN4*, and *TMT* opsins was detected in RT-PCR analysis, we cannot completely rule out the possibility that other types of opsins are involved in chromatophore photoresponses represented herein. The expression of non-visual opsins sensitive to UV and green light has been identified in photosensitive organs of different vertebrates [60]–[63]. For example, evidence has shown a non-mammalian type of UV-sensitive neuropsin (cOPN5L2) in birds [61] and green-sensitive vertebrate ancient (VA) opsins in zebrafish [62], [63]. Because of the diversity of non-rod, non-cone visual pigments and their unclear functions in various organs, more molecular data and functional analysis are required to exclude their possible involvement in chromatophore photoresponses. Furthermore, the details of how photoreception and phototransduction take place within chromatophores are unknown so far. There is not yet direct evidence that opsins are expressed in cell membrane, cytoplasm, or intracellular granules. It is also unclear whether the opsins utilize the identical type of the light-sensitive chromophore in retina, although chromophores were isolated from extraretinal photoreceptors in some species [64]–[66]. It was suggested that non-image-forming photosensitive systems may adopt the chromophore selected by the visual system [65]. In order to understand if ipDCs compose a dermal photosensory system that independently detect a change in quantity and quality of light, expression studies alone are not enough and more cellular evidence and functional analysis are required, especially on under-characterized photosensitive systems.

### Conclusions

Our data demonstrate opsin expression in tilapia integumentary tissue. More specifically, we identify and describe the fundamental molecular information of co-expression of opsins within individual chromatophores, which could be related to distinct photoresponses of different types of ipDCs. These observations indicate that diverse molecular mechanisms and photoreactive strategies may be employed by different classes of chromatophores, which play distinctive roles in pigment pattern formation and other physiological functions [53], [67], [68]. In the future, we will analyze chromatic performance of chromatophores to characterize the spectral sensitivity. This information will not only help us perform a comprehensive comparison of chromatic interactions of photoreceptors/opsins in retina and extraretinal tissues, but it will also provide an excellent chance to understand how animals coordinate multiple visual cues from different light-sensitive organs.

## Supporting Information

Movie S1 **Melanophore photoresponses under illumination.** Melanophore photoresponses under illumination at 500 nm (13.9 log photons cm^−2^ s^−1^) for 10 minutes. Under illuminations, melanophores tended to maintain their shape in the dispersion stage by shuttling pigment granules (melanosomes). Scale bars: 20 µm.
(WMV)

## References

[1] Auerswald L, Freier U, Lopata A, Meyer B (2008) Physiological and morphological colour change in Antarctic krill, *Euphausia superba*: a field study in the Lazarev Sea. J Exp Biol 211: 3850–3858. 19043057 10.1242/jeb.024232

[2] Fuhrmann MM, Nygard H, Krapp RH, Berge J, Werner I (2011) The adaptive significance of chromatophores in the Arctic under-ice amphipod *Apherusa glacialis* . Polar Biol 34: 823–832.

[3] de Velasco JB, Tattersall GJ (2008) The influence of hypoxia on the thermal sensitivity of skin colouration in the bearded dragon, *Pogona vitticeps* . J Comp Physiol B 178: 867–875. 18491114 10.1007/s00360-008-0274-8

[4] Silbiger N, Munguia P (2008) Carapace color change in *Uca pugilator* as a response to temperature. J Exp Mar Biol Ecol 355: 41–46.

[5] Chiou TH, Mäthger LM, Hanlon RT, Cronin TW (2007) Spectral and spatial properties of polarized light reflections from the arms of squid (*Loligo pealeii*) and cuttlefish (*Sepia officinalis L.*). J Exp Biol 210: 3624–3635. 17921164 10.1242/jeb.006932

[6] Shashar N, Rutledge PS, Cronin TW (1996) Polarization vision in cuttlefish - A concealed communication channel? J Exp Biol 199: 2077–2084. 9319987 10.1242/jeb.199.9.2077

[7] Chiao CC, Chubb C, Buresch KC, Barbosa A, Allen JJ, et al (2010) Mottle camouflage patterns in cuttlefish: quantitative characterization and visual background stimuli that evoke them. J Exp Biol 213: 187–199. 20038652 10.1242/jeb.030247

[8] Hanlon RT, Messenger JB (1988) Adaptive Coloration in Young Cuttlefish (*Sepia-Officinalis L*) - the Morphology and Development of Body Patterns and Their Relation to Behavior. Philos Trans R Soc B-Biol Sci 320: 437–487.

[9] Skold HN, Amundsen T, Svensson PA, Mayer I, Bjelvenmark J, et al (2008) Hormonal regulation of female nuptial coloration in a fish. Horm Behav 54: 549–556. 18586039 10.1016/j.yhbeh.2008.05.018

[10] Svensson PA, Forsgren E, Amundsen T, Skold HN (2005) Chromatic interaction between egg pigmentation and skin chromatophores in the nuptial coloration of female two-spotted gobies. J Exp Biol 208: 4391–4397. 16339859 10.1242/jeb.01925

[11] Muske LE, Fernald RD (1987) Control of a teleost social signal. I. Neural basis for differential expression of a color pattern. J Comp Physiol A 160: 89–97. 3820134 10.1007/BF00613444

[12] Pauers MJ, Kapfer JM, Fendos CE, Berg CS (2008) Aggressive biases towards similarly coloured males in Lake Malawi cichlid fishes. Biol Lett 4: 156–159. 18230585 10.1098/rsbl.2007.0581 PMC2429924

[13] Lanzing WJR, Bower CC (1974) Development of Color Patterns in Relation to Behavior in *Tilapia Mossambica* (Peters). J Fish Biol 6: 29–41.

[14] Muske LE, Fernald RD (1987) Control of a teleost social signal. II. Anatomical and physiological specializations of chromatophores. J Comp Physiol A 160: 99–107. 3820135 10.1007/BF00613445

[15] Fujii R (1993) Cytophysiology of fish chromatophores. International Review of Cytology 143: 191–255.

[16] Fujii R (2000) The regulation of motile activity in fish chromatophores. Pigment Cell Res 13: 300–319. 11041206 10.1034/j.1600-0749.2000.130502.x

[17] Goda M, Fujii R (1995) Blue chromatophores in two species of callionymid fish. Zool Sci 12: 811–813.

[18] Hatamoto K, Shingyoji C (2008) Cyclical training enhances the melanophore responses of zebrafish to background colours. Pigment Cell Melanoma Res 21: 397–406. 18476910 10.1111/j.1755-148X.2008.00445.x

[19] Sugimoto M, Uchida N, Hatayama M (2000) Apoptosis in skin pigment cells of the medaka, *Oryzias latipes* (Teleostei), during long-term chromatic adaptation: the role of sympathetic innervation. Cell Tissue Res 301: 205–216. 10955716 10.1007/s004410000226

[20] Bagnara JT, Taylor JD, Hadley ME (1968) Dermal Chromatophore Unit. J Cell Biol 38: 67–79. 5691979 10.1083/jcb.38.1.67 PMC2107474

[21] Grether GF, Kolluru GR, Nersissian K (2004) Individual colour patches as multicomponent signals. Biol Rev Camb Philos Soc 79: 583–610. 15366764 10.1017/s1464793103006390

[22] Kashina A, Rodionov V (2005) Intracellular organelle transport: few motors, many signals. Trends Cell Biol. 15: 396–398. 10.1016/j.tcb.2005.06.002 16005630

[23] Rodionov V, Yi J, Kashina A, Oladipo A, Gross SP (2003) Switching between microtubule- and actin-based transport systems in melanophores is controlled by cAMP levels. Curr Biol 13: 1837–1847. 14588239 10.1016/j.cub.2003.10.027

[24] Burton D (2008) A physiological interpretation of pattern changes in a flatfish. J Fish Biol 73: 639–649.

[25] Pye JD (1964) Nervous Control of Chromatophores in Teleost Fishes. I. Electrical Stimulation in Minnow (*Phoxinus Phoxinus* (L.)). J Exp Biol 41: 525–534.

[26] Iga T, Takabatake I (1982) Action of melanophore-stimulating hormone on melanophores of the cyprinid fish *Zacco temmincki* . Comp Biochem Physiol C 73: 51–55. 6128180 10.1016/0306-4492(82)90167-8

[27] Nagaishi H, Oshima N (1989) Neural control of motile activity of light-sensitive iridophores in the neon tetra. Pigment Cell Res 2: 485–492. 2557604 10.1111/j.1600-0749.1989.tb00243.x

[28] Oshima N, Makino M, Iwamuro S, Bern HA (1996) Pigment dispersion by prolactin in cultured xanthophores and erythrophores of some fish species. J Exp Zool 275: 45–52.

[29] Oshima N, Nakamaru N, Araki S, Sugimoto M (2001) Comparative analyses of the pigment-aggregating and -dispersing actions of MCH on fish chromatophores. Comp Biochem Physiol C Toxicol Pharmacol 129: 75–84. 11423380 10.1016/s1532-0456(01)00187-9

[30] van der Salm AL, Metz JR, Bonga SE, Flik G (2005) Alpha-MSH, the melanocortin-1 receptor and background adaptation in the Mozambique tilapia, *Oreochromis mossambicus* . Gen Comp Endocrinol 144: 140–149. 16019002 10.1016/j.ygcen.2005.05.009

[31] Nery LE, Castrucci AM (1997) Pigment cell signalling for physiological color change. Comp Biochem Physiol A Physiol 118: 1135–1144. 9505423 10.1016/s0300-9629(97)00045-5

[32] Ban E, Kasai A, Sato M, Yokozeki A, Hisatomi O, et al (2005) The signaling pathway in photoresponses that may be mediated by visual pigments in erythrophores of Nile tilapia. Pigment Cell Res 18: 360–369. 16162176 10.1111/j.1600-0749.2005.00267.x

[33] Kasai A, Oshima N (2006) Light-sensitive motile iridophores and visual pigments in the neon tetra, *Paracheirodon innesi.* . Zool Sci 23: 815–819. 17043404 10.2108/zsj.23.815

[34] Oshima N, Yokozeki A (1999) Direct Control of Pigment Aggregation and Dispersion in Tilapia Erythrophores by Light. Zool Sci 16: 51–54.

[35] Sato M, Ishikura R, Oshima N (2004) Direct effects of visible and UVA light on pigment migration in erythrophores of Nile tilapia. Pigment Cell Res 17: 519–524. 15357839 10.1111/j.1600-0749.2004.00178.x

[36] Naora H, Takabatake I, Iga T (1988) Spectral sensitivity of melanophores of a freshwater teleost, *Zacco temmincki* . Comp Biochem Physiol A 90: 147–149. 2900099 10.1016/0300-9629(88)91020-1

[37] Negishi S (1985) Light response of cultured melanophores of a teleost adult fish, *Oryzias latipes* . J Exp Zool 236: 327–333.

[38] Obika M, Meyer-Rochow VB (1990) Dermal and epidermal chromatophores of the Antarctic teleost *Trematomus bernacchii* . Pigment Cell Res 3: 33–37. 2377579 10.1111/j.1600-0749.1990.tb00259.x

[39] Oshima N, Nakata E, Ohta M, Kamagata S (1998) Light-induced pigment aggregation in xanthophores of the medaka, *Oryzias latipes* . Pigment Cell Res 11: 362–367. 9870548 10.1111/j.1600-0749.1998.tb00495.x

[40] Lythgoe JN, Shand J, Foster RG (1984) Visual pigment in fish iridocytes. Nature 308: 83–84.

[41] Oshima N (2001) Direct reception of light by chromatophores of lower vertebrates. Pigment Cell Res 14: 312–319. 11601652 10.1034/j.1600-0749.2001.140502.x

[42] Yokoyama S (2000) Molecular evolution of vertebrate visual pigments. Prog. Retin. Eye Res. 19: 385–419. 10.1016/s1350-9462(00)00002-1 10785616

[43] Spady TC, Parry JWL, Robinson PR, Hunt DM, Bowmaker JK, et al (2006) Evolution of the cichlid visual palette through ontogenetic subfunctionalization of the opsin gene arrays. Mol Biol Evol 23: 1538–1547. 16720697 10.1093/molbev/msl014

[44] Halstenberg S, Lindgren KM, Samagh SPS, Nadal-Vicens M, Balt S, et al (2005) Diurnal rhythm of cone opsin expression in the teleost fish *Haplochromis burtoni* . Visual Neurosci 22: 135–141. 10.1017/S0952523805222022 15935106

[45] Masagaki A, Fujii R (1999) Differential actions of melatonin on melanophores of the threeline pencilfish, *Nannostomus trifasciatus* . Zool Sci 16: 35–42.

[46] Fujii R, Wakatabi H, Oshima N (1991) Inositol 1,4,5-Trisphosphate Signals the Motile Response of Fish Chromatophores. I. Aggregation of Pigment in the Tilapia Melanophore. J Exp Zool 259: 9–17.

[47] Masada M, Matsumoto J, Akino M (1990) Biosynthetic pathways of pteridines and their association with phenotypic expression in vitro in normal and neoplastic pigment cells from goldfish. Pigment Cell Res 3: 61–70. 2201016 10.1111/j.1600-0749.1990.tb00324.x

[48] Oshima N, Suzuki M, Yamaji N, Fujii R (1988) Pigment Aggregation Is Triggered by an Increase in Free Calcium-Ions within Fish Chromatophores. Comp Biochem Physiol A Physiology 91: 27–32.

[49] Sugimoto M, Yuki M, Miyakoshi T, Maruko K (2005) The influence of long-term chromatic adaptation on pigment cells and striped pigment patterns in the skin of the zebrafish, *Danio rerio* . J Exp Zool A 303: 430–440. 10.1002/jez.a.177 15880775

[50] Sugimoto M, Oshima N (1995) Changes in adrenergic innervation to chromatophores during prolonged background adaptation in the medaka, *Oryzias latipes* . Pigment Cell Res 8: 37–45. 7792253 10.1111/j.1600-0749.1995.tb00772.x

[51] Daniolos A, Lerner AB, Lerner MR (1990) Action of light on frog pigment cells in culture. Pigment Cell Res 3: 38–43. 2165596 10.1111/j.1600-0749.1990.tb00260.x

[52] Moriya T, Miyashita Y, Arai J, Kusunoki S, Abe M, et al (1996) Light-sensitive response in melanophores of *Xenopus laevis*: I. Spectral characteristics of melanophore response in isolated tail fin of *Xenopus* tadpole. J Exp Zool 276: 11–18. 8828182 10.1002/(SICI)1097-010X(19960901)276:1<11::AID-JEZ2>3.0.CO;2-8

[53] Armstrong TN, Cronin TW, Bradley BP (2000) Microspectrophotometric analysis of intact chromatophores of the Japanese medaka, *Oryzias latipes* . Pigment Cell Res 13: 116–119. 10841033 10.1034/j.1600-0749.2000.130210.x

[54] Hofer R, Mokri C (2000) Photoprotection in tadpoles of the common frog, *Rana temporaria* . J Photochem Photobiol B-Biol 59: 48–53. 10.1016/s1011-1344(00)00136-6 11332890

[55] Shand J, Foster RG (1999) The extraretinal photoreceptors of non-mammalian vertebrates. In: Archer SN, Djamgoz MBA, Leow ER, Partridge JC, Vallerga S, editors. Adaptive Mechanisms in the Ecology of Vision: Kluwer Academic Publishers. 197–222.

[56] Tosini G, Avery RA (1996) Dermal photoreceptors regulated basking behavior in the lizard *Podarcis muralis* . Physiol Behav 59: 195–198. 8848482 10.1016/0031-9384(95)02040-3

[57] Ullrich-Luter EM, Dupont S, Arboleda E, Hausen H, Arnone MI (2011) Unique system of photoreceptors in sea urchin tube feet. Proc Natl Acad Sci USA 108: 8367–8372. 21536888 10.1073/pnas.1018495108 PMC3100952

[58] Yerramilli D, Johnsen S (2010) Spatial vision in the purple sea urchin *Strongylocentrotus purpuratus* (Echinoidea). J Exp Biol 213: 249–255. 20038658 10.1242/jeb.033159

[59] Forsell J, Ekstrom P, Flamarique IN, Holmqvist B (2001) Expression of pineal ultraviolet- and green-like opsins in the pineal organ and retina of teleosts. J Exp Bio 204: 2517–2525. 11511667 10.1242/jeb.204.14.2517

[60] Wada S, Kawano-Yamashita E, Koyanagi M, Terakita A (2012) Expression of UV-sensitive parapinopsin in the iguana parietal eyes and its implication in UV-sensitivity in vertebrate pineal-related organs. PLoS ONE 7: e39003. 22720013 10.1371/journal.pone.0039003 PMC3375259

[61] Ohuchi H, Yamashita T, Tomonari S, Fujita-Yanagibayashi S, Sakai K, et al (2012) A non-mammalian type opsin 5 functions dually in the photoreceptive and non-photoreceptive organs of birds. PLoS ONE 7: e31354. 22348098 10.1371/journal.pone.0031534 PMC3279408

[62] Kojima D, Torii M, Fukada Y, Dowling JE (2008) Differential expression of duplicated VAL-opsin genes in the developing zebrafish. J Neurochem 104: 1364–1371. 18036148 10.1111/j.1471-4159.2007.05093.x PMC2702163

[63] Kojima D, Mano H, Fukada Y (2000) Vertebrate ancient-long opsin: a green-sensitive photoreceptive molecule present in zebrafish deep brain and retinal horizontal cells. J Neurosci 20: 2845–2851. 10751436 10.1523/JNEUROSCI.20-08-02845.2000 PMC6772192

[64] Foster RG, Garciafernandez JM, Provencio I, Degrip WJ (1993) Opsin Localization and Chromophore Retinoids Identified within the Basal Brain of the Lizard *Anolis-Carolinensis* . J Comp Physiol A 172: 33–45.

[65] Provencio I, Foster RG (1993) Vitamin A2-based photopigments within the pineal gland of a fully terrestrial vertebrate. Neurosci Lett 155: 223–226. 8377952 10.1016/0304-3940(93)90713-u

[66] Tabata M, Suzuki T, Niwa H (1985) Chromophores in the Extraretinal Photoreceptor (Pineal Organ) of Teleosts. Brain Res 338: 173–176. 4027587 10.1016/0006-8993(85)90263-x

[67] Fletcher TC (1978) Defense mechanisms in fish. In: Malins DC, Sargent JR, editors. Biochemical and Biophysical Perspectives in Marine Biology. London: Academic Press. 189–217.

[68] Kelsh RN, Harris ML, Colanesi S, Erickson CA (2009) Stripes and belly-spots-A review of pigment cell morphogenesis in vertebrates. Semin Cell Dev Biol 20: 90–104. 18977309 10.1016/j.semcdb.2008.10.001 PMC2744437

